# APP as a Protective Factor in Acute Neuronal Insults

**DOI:** 10.3389/fnmol.2017.00022

**Published:** 2017-02-02

**Authors:** Dimitri Hefter, Andreas Draguhn

**Affiliations:** ^1^Institute of Physiology and Pathophysiology, Heidelberg UniversityHeidelberg, Germany; ^2^Department of Psychiatry and Psychotherapy, Central Institute of Mental Health, Medical Faculty Mannheim, Heidelberg UniversityMannheim, Germany

**Keywords:** Alzheimer, ischemia, calcium toxicity, cell death, amyloid precursor protein, stroke, traumatic brain injury, neuroprotection

## Abstract

Despite its key role in the molecular pathology of Alzheimer’s disease (AD), the physiological function of amyloid precursor protein (APP) is unknown. Increasing evidence, however, points towards a neuroprotective role of this membrane protein in situations of metabolic stress. A key observation is the up-regulation of APP following acute (stroke, cardiac arrest) or chronic (cerebrovascular disease) hypoxic-ischemic conditions. While this mechanism may increase the risk or severity of AD, APP by itself or its soluble extracellular fragment APPsα can promote neuronal survival. Indeed, different animal models of acute hypoxia-ischemia, traumatic brain injury (TBI) and excitotoxicity have revealed protective effects of APP or APPsα. The underlying mechanisms involve APP-mediated regulation of calcium homeostasis via NMDA receptors (NMDAR), voltage-gated calcium channels (VGCC) or internal calcium stores. In addition, APP affects the expression of survival- or apoptosis-related genes as well as neurotrophic factors. In this review, we summarize the current understanding of the neuroprotective role of APP and APPsα and possible implications for future research and new therapeutic strategies.

## Introduction

Amyloid precursor protein (APP) has been first described in 1987 as a potential substrate of pathological deposits in the nervous system (Kang et al., 1987). By now, there is good evidence from multiple lines of research that specific domains of APP do indeed contribute to amyloid plaques as found in patients with Alzheimer’s disease (AD; Hardy and Selkoe, 2002). On the other hand, the function of this ubiquitously expressed protein in healthy brains remains poorly understood. Recent evidence from neurological patients and from different disease models hint towards a potential neuroprotective function of APP under conditions of acute cellular insult: APP is up-regulated following hypoxia, ischemia or traumatic brain injury (TBI; Van den Heuvel et al., 1999; Pottier et al., 2012). This reaction coincides well with some known interactions between APP and other proteins which are relevant for homeostatic regulation of cell integrity under stressful conditions, such as certain glutamate receptors, calcium channels or gene-regulatory networks (Russo et al., 2005). With respect to the underlying molecular mechanisms it is important to note that the integral membrane protein APP can give rise to both, protective and potentially damaging molecules following cleavage by different secretases (Brunholz et al., 2012). These cleavage processes keep a balance between different amyloidogenic and non-amyloidogenic products of APP, including the protective APPsα fragment which is secreted into the extracellular space (Mattson et al., 1993a). Together, APP or its fragments may well have a neuroprotective role during acute challenges of neuronal integrity, and it may exert this function by regulating neuronal calcium homeostasis and cell survival. Novel findings on APP-related neuroprotective mechanisms open promising new therapeutic strategies in stroke, AD and TBI.

In the present review article, we summarize the evidence for a neuroprotective function of APP in the adult brain. After a brief introduction of the protein and its metabolites, we summarize current knowledge from clinical, animal and *in vitro* studies about its role in stroke, brain injury and neurodegeneration. Finally, we discuss possible mechanisms and point out several promising therapeutic targets.

## APP Structure, Expression, Trafficking, Cleavage and Subcellular Localization

APP is a type-1 transmembrane protein comprising a long extracellular N-terminal domain, a transmembrane region and an intracellular C-terminal domain, APP intracellular domain (AICD; Kang et al., 1987). Alternative splicing of the APP gene, which is located on chromosome 21, produces three isoforms containing 695, 751 and 770 amino acids, respectively (Beyreuther et al., 1993). While APP751 and APP770 are expressed almost ubiquitously, APP695 can be found nearly exclusively in neurons. Depending on the isoform, the APP extracellular domain consists of up to six different subdomains with specific structural motives and various binding partners such as extracellular matrix proteins (heparine, collagene, laminine, proteoglycans), metals (copper, zinc) and regulatory proteins (LDL-receptor-related protein, F-spondin; Gralle and Ferreira, 2007; Müller and Zheng, 2012). After translation in the endoplasmic reticulum (ER), APP undergoes various post-translational modifications in the Golgi complex before it is transported to the cell membrane (Caporaso et al., 1994). The mature membrane protein can be processed by different membrane-associated proteolytic enzymes, beginning with cleavage of the transmembrane domain by γ-secretase. Subsequent cleavage by α-secretase results in three fragments: AICD, a short p3 fragment and the secreted soluble APP α (APPsα). Alternatively, cleavage by the β-secretase BACE-1 (Beta-site APP Cleaving Enzyme 1) releases APPsβ and the neurotoxic amyloid β (Aβ) peptide (refer to Haass et al., 2012 for review on processing of APP). Under normal conditions, only a small fraction of the expressed APP is secreted, and cleavage by α-secretases outweighs the amyloidogenic pathway by far (Hick et al., 2015).

In neurons, APP is found in somatodendritic and axonal compartments as well as in the presynaptic active zone (Laßek et al., 2016) which it reaches by fast axonal transport (Brunholz et al., 2012). Its intracellular trafficking involves four different neuronal trafficking adaptors including Mint1 and is regulated by tyrosine phosphorylation (Dunning et al., 2016). Expression, trafficking and processing of APP are complexly regulated, including prominent changes during pathological states. APP expression is upregulated under conditions of metabolic stress (Hoyer et al., 2005), ischemia (Pottier et al., 2012), brain injury (Van den Heuvel et al., 1999) and inflammation (Herbst-Robinson et al., 2015). APP processing and degradation differ under conditions of acute stress. In response to increased levels of intracellular calcium, APP is degraded via the ubiquitin-proteasome proteolytic pathway (Jung et al., 2015). Facilitated degradation might counteract overexpression of APP under conditions of acute stress, prevent accumulation of misfolded protein and its processing into Aß. As an additional adaptive mechanism, cleavage of the protein is regulated by synaptic activity, affecting the balance between amyloidogenic and non-amyloidogenic pathways (Kamenetz et al., 2003; Cirrito et al., 2005). Intriguingly, APP is expressed and cleaved heterogeneously in different types of neurons and in astrocytes and in different brain areas, which might contribute to variable susceptibility to insults between brain regions and cell types (Del Turco et al., 2016; Liao et al., 2016). Activated by proinflammatory cytokines, astrocytes were shown both to contribute to Aß production as well as to stimulate the secretion of APPsα, suggesting a significant contribution of glia cells to production and cleavage of APP and a tight coupling between APP processing and the immune system (Zhao et al., 2011; Yang et al., 2015). While still quite superficially understood, this activity- and stress-dependent multi-level relation of APP in neural, glial and immune cell response strongly suggests a role as an acute phase protein with functions in cellular survival under metabolically challenging conditions.

## Functions of APP and Its Metabolites

APP is highly conserved across different phyla including mammals, insects and nematodes, suggesting that the protein has advantageous effects on survival and reproduction of animals (Müller and Zheng, 2012; van der Kant and Goldstein, 2015). Indeed, in the nematode *C. elegans* knock-out of APP-like protein (APL-1) is lethal (Hornsten et al., 2007). *Drosophila* lacking the APP ortholog APPL exhibit severe memory deficits (Bourdet et al., 2015). Most knowledge on systemic functions of APP has been gained from studies of genetically modified rodents. Remarkably, mice lacking APP are viable, fertile, and exhibit a relatively mild phenotype. Alterations include reduced body and brain weight and several neurological symptoms like reduced grip strength (Weyer et al., 2011; Caldwell et al., 2013), deficits in spatial memory (Puzzo et al., 2011), and increased susceptibility to seizures (Steinbach et al., 1998). This phenotype may be related to changes at the cellular and network level like reduced numbers of dendritic spines, reduced hippocampal LTP and altered short-term plasticity (Seabrook et al., 1999; Weyer et al., 2011; Jedlicka et al., 2012; Korte et al., 2012). The absence of more severe deficits is likely due to the existence of homologous proteins, called APLP1 and APLP2, which can compensate the lack of APP due to overlapping functions (Aydin et al., 2012). Indeed, double knockout mice lacking two of the three homologous proteins are much more heavily affected: mice lacking APP and the globally expressed APLP2 as well as APLP1-KO/APLP2-KO mice die perinatally due to impaired neuromuscular transmission (Wang et al., 2005), while mice deficient for APP and APLP1, which is predominantly expressed in the brain, survive birth but exhibit rather severe deficits (Heber et al., 2000). Triple knock-out mice die during embryonic development or shortly after birth and show lissencephaly-like cortical malformations (Herms et al., 2004), pointing towards a role for APP and its homologs in essential developmental mechanisms like neuronal migration, neurite outgrowth and synaptogenesis. Detailed studies at the cellular and molecular level revealed several further functions of APP. The protein is involved in regulation of synaptic vesicle exocytosis (Kohli et al., 2012) glutamatergic, GABAergic and cholinergic synaptic transmission (Wang et al., 2005, 2014; Schrenk-Siemens et al., 2008) and synapse formation (Priller et al., 2006). Interestingly, it also regulates endosomal phosphoinositide metabolism and prevents neurodegeneration (Balklava et al., 2015), and it interacts with a large variety of survival-related cascades (Russo et al., 2005; Venezia et al., 2007).

### APPsα and APPsβ

Several functions of APP seem to be mediated by its soluble cleavage product APPsα. Selective expression of APPsα in mice with APP^−/−^ background abolishes most of their deficits, rescuing LTP as well as the typical anatomical and behavioral abnormalities (Ring et al., 2007; Hick et al., 2015). Mice selectively expressing APPsα on APP-KO/APLP2-KO background (which by itself is lethal) survive well into adulthood and show only a mildly altered phenotype, similar to simple APP-KO animals (Zhang et al., 2013). Enhancing APPsα levels by over-expression of ADAM-10 increases cortical synaptogenesis *in vivo* (Bell et al., 2008). Intraventricular application of APPsα enhances memory in mice (Meziane et al., 1998). Altogether, there is strong evidence that APPsα mediates many of the effects of APP on brain development and supports several cognitive functions. In addition, the APPsα fragment has been shown to mediate a variety of neuroprotective and trophic effects (Hick et al., 2015; Fol et al., 2016; Hefter et al., 2016; Plummer et al., 2016), as discussed in following sections. It is important to note that secretion of APPsα is regulated by neuronal activity (Kirazov et al., 1997; Gakhar-Koppole et al., 2008) and by activated astrocytes (Yang et al., 2015). This may point towards state-dependent functions of the protein, in line with the neuroprotective effects described below. The trophic effects of APPsα are dose-dependent, beginning as low as 100 pM, reaching an optimum at 10 nM and decreasing at higher doses (Demars et al., 2011).

Notably, APPsβ fails to mimic the beneficial effects of APPsα, although there is only a difference of 16 amino acids between both proteins (Hick et al., 2015). In other studies, however, trophic effects of APPsβ were detected, albeit less potent than those of APPsα (Chasseigneaux et al., 2011). Interestingly, APPsβ was found to undergo further proteolytic cleavage and bind to “death receptor 6”, activating caspase-6 and thus contributing to neurodegeneration (Nikolaev et al., 2009).

### APP Intracellular Domain (AICD)

The intracellular domain of APP, termed AICD, interacts with various cytosolic signaling cascades including glycogen synthase kinase 3 (GSK-3), Ras proteins and MAPK pathways and is able to translocate to the nucleus after forming a complex with the adaptor protein Fe65 (Schettini et al., 2010). There, it is involved in regulation of genes associated with survival and apoptosis (Müller et al., 2008; Multhaup et al., 2015). Indeed, overexpression of AICD was found to induce apoptosis by interaction with the p53-pathway (Ozaki et al., 2006; Nakayama et al., 2008). Moreover, AICD modulates intracellular calcium homeostasis and ATP content (Hamid et al., 2007) and affects synaptic plasticity and hippocampus-dependent learning by increasing LTP (Klevanski et al., 2015).

### Amyloid ß

Resulting from APP cleavage by BACE-1, Aβ peptides can accumulate extracellularly as soluble oligomers or in amyloid plaques, promoting neurodegeneration in AD (Haass, 2010). Interestingly, Aβ can be internalized by neurons and accumulates in the cytosol as well as within endosomes/lysosomes and mitochondria (Chen and Yan, 2006). It exerts neurotoxic effects via a variety of mechanisms, such as disruption of calcium homeostasis (Berridge, 2010), overactivation of mGluR5 (Zhang et al., 2015), impairment of synaptic transmission, plasticity and network function (Kamenetz et al., 2003; Palop and Mucke, 2010), mitochondrial dysfunction (Chen and Zhong, 2013) and apoptosis (Umeda et al., 2011). Remarkably, it is also able to translocate into the nucleus and influence apoptosis-related gene transcription (Barucker et al., 2014; Multhaup et al., 2015). The APP fragment has also been suggested to form channel-like pores in neuronal membranes, but the underlying mechanisms are currently unknown (Barucker et al., 2014).

## Links between Ischemia, Brain Injury and Neurodegeneration—Results from Human Studies

Sporadic AD is the most common cause of dementia and constitutes one of the most imminent medical problems in developed countries (Prince et al., 2015). Cognitive deficits in AD are caused by progressive loss of neurons, beginning in the temporal lobe and resulting in severe global brain atrophy (Fox and Schott, 2004). The neuronal loss is linked to pathological accumulation of amyloid and tau protein, as first described by Alzheimer (1906). No causal treatments exist at this stage of the disease. However, irreversible macroscopic pathology and cognitive decline are preceded by functional deficits such as disturbance of cellular calcium- and energy-homeostasis (Mattson, 1994), mitochondrial dysfunction (Swerdlow and Khan, 2004; Rönnbäck et al., 2016), synaptic failure (Selkoe, 2002) and activation of pro-apoptotic pathways (Mattson, 2000), offering an opportunity for detection and intervention at the preclinical stage (Jack and Holtzman, 2013). Interestingly, various lines of evidence suggest that molecular pathomechanisms in AD such as amyloid deposition and disrupted calcium homeostasis overlap with those in hypoxia-ischemia (Peers et al., 2009) and TBI (Magnoni and Brody, 2010).

In many pathologies of the CNS such as TBI and stroke the brain-blood barrier (BBB) is disturbed which results in extravasation of blood-derived proteins including albumin and inflammatory mediators into the brain tissue (Zhao et al., 2015). Presence of albumin in the extracellular space may act epileptogenic and promote degeneration (Friedman et al., 2009). Inflammatory cytokines may regulate secretase activity and both facilitate non-amyloidogenic cleavage as well as Aß deposition (Zhao et al., 2011; Yang et al., 2015), possibly contributing to the development of AD later on (Sastre et al., 2008). Alternatively, amyloid may directly diffuse from vessels into the brain tissue through a malfunctioning BBB (Pluta et al., 2009). Amyloid plaques, in turn, are well known to evoke a strong inflammatory response with activation of microglia, astrocytes and inflammatory mediators.

Amyloid accumulates and deposits into plaques if its intracellular degradation and extracellular clearance are disturbed. Proteolytic degradation is inhibited by lack of energy substrates and oxidative stress, while extracellular degradation requires intact interstitial and cerebrospinal fluid flow and BBB function and is impaired in inflammation (Iliff et al., 2015; Tarasoff-Conway et al., 2015). As these processes are disturbed in stroke and TBI, both conditions may lead to impaired amyloid clearance and AD development.

**Figure 1:**
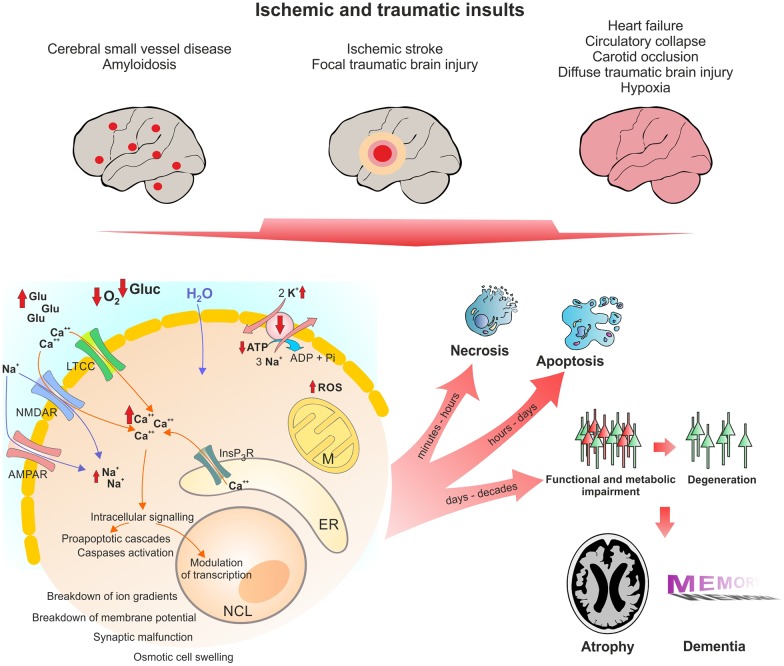
**Pathophysiological changes in neurons following acute ischemic and traumatic insults.** Micro- and macroscopic focal strokes, global hypoxia-ischemia and traumatic brain injury (TBI) lead to abruption of extracellular glucose and oxygen supply and excessive glutamate release. One major shared pathomechanism is NMDAR-mediated excitotoxicity, or over-activation of NMDAR by glutamate, which facilitates sodium and calcium influx. Due to excessive ion influx, the cellular membrane potential is depolarized, which leads to activation of voltage gated calcium channels such as LTCC, initiating a vicious cycle of ion influx, calcium overload, depolarization and aberrant activity. Successively calcium from intracellular calcium stores, particularly mitochondria and the ER, is released, increasing calcium levels to up to 200-fold of ~100 nM during resting. Calcium activates secondary messengers that are able to translocate to the nucleus and modulate gene transcription. Long-lasting or severely elevated calcium levels may lead to activation of caspases and apoptosis. Following the osmotic gradient, water enters the cell and leads to cell swelling and brain edema. Due to glucose and oxygen shortage and excessive formation of reactive oxygen species (ROS), mitochondrial function is compromised and ATP production halts. Malfunction of the energy demanding ion pumps, predominantly the sodium potassium pump, ultimately leads to breakdown of the membrane potential, a phenomenon known as anoxic or hypoxic spreading depolarization or spreading depression (due to depression of network activity in the field potential recording). Given the energy supply is timely restored, this stage can be reversed without long-lasting morphological damage. If the insult is protracted, neurons might undergo (dependent of insult’s severity) necrotic or apoptotic death or degenerate with a delay of days to decades due to synaptic or metabolic malfunction. Acute cell death and delayed degeneration contribute to brain atrophy and development of dementia. Glu, glutamate; Gluc, glucose; LTCC, L-Type calcium channel; NMDAR, NMDA receptor; AMPAR, AMPA receptor; M, mitochondrion; NCL, nucleus; ER, endoplasmic reticulum; ROS, reactive oxygen species.

In line with these pathomechanisms, a history of TBI (Fleminger et al., 2003; Sivanandam and Thakur, 2012), stroke (Thiel et al., 2014) and cardiac arrest (de la Torre, 2006) are risk factors for developing AD. Below, we will describe the similarities between these conditions in detail. Figure 1 shows the pathophysiological cascades leading from acute insult to long-term neurodegeneration.

## Traumatic Brain Injury, APP and AD

### TBI Leads to Amyloid Pathology and Strongly Increases the Risk for AD and Cognitive Decline

TBI is a debilitating and life-threatening condition which is the leading cause of disability in people under 35 years in industrial countries (Feigin et al., 2013). Besides acute primary damage, TBI promotes secondary neurodegeneration and increases the risk for developing AD by ~2-fold (Mortimer et al., 1991; Mayeux et al., 1993; Schofield et al., 1997; Guo et al., 2000; Fleminger et al., 2003). Following TBI, diffuse Aβ deposits can be found in the temporal cortex as early as 2 h after the insult (Ikonomovic et al., 2004). Furthermore, post mortem histological analysis shows that deposition of amyloid ß-protein in the brain occurs in approximately one-third of individuals who die shortly after a severe head injury (Roberts et al., 1994). Aβ levels are altered in cerebro-spinal and interstitial cerebral fluid in patients with TBI (Magnoni and Brody, 2010; Tsitsopoulos and Marklund, 2013) and correlate with clinical outcome (Magnoni and Brody, 2010). A history of TBI prior to the onset of dementia correlates with greater amyloid burden in patients with mild cognitive deficits (Mielke et al., 2014) and is associated with faster rates of cognitive decline in AD patients (Moretti et al., 2012; Gilbert et al., 2014). In TBI patients, APP transcription is upregulated and its axonal transport is interrupted due to diffuse axonal injury, which results in deposition of APP and its products in axonal “bulbs” (Hayashi et al., 2015). These results from human studies are in line with a large body of evidence from various models of TBI in mice, rats and sheep, where APP overexpression following TBI has been extensively studied. In models of focal cerebral injury inflicted by stabbing or weight drop local APP immunoreactivity increased in neurons as well as in astrocytes (Otsuka et al., 1991; Lewén et al., 1995, 1996). In the midline fluid percussion model of diffuse brain injury in adult rats APP expression was globally elevated in cortex and hippocampus within hours following the insult (Murakami et al., 1998); in a lateral fluid percussion model APP was overexpressed as early as 1 h after the insult (Pierce et al., 1996). In a weight fall model of brainstem injury in adult rats APP mRNA levels rose as soon as 1 h post-impact, peaked 3 h after the injury at almost twofold baseline level and declined to baseline within 24 h (Yang et al., 2014). Similarly, in an ovine TBI model APP mRNA was up-regulated as soon as 30 min post-impact (Van den Heuvel et al., 1999).

### Protective Function of APP and APPsα in TBI

While over-expression of APP following mechanical insults has been observed several decades ago, the functional effects remained unclear until recently. By now, evidence from different animal models points towards an acute neuroprotective effect of APP and APPsα in TBI (Plummer et al., 2016). In diffuse traumatic injury in rats, intraventricular administration of APPsα 30 min after the insult reduced axonal injury and apoptosis and improved motor and cognitive outcome (Thornton et al., 2006). In the same model of TBI, mice lacking APP suffered from greater cognitive and motor impairment in correspondence with larger lesions and increased hippocampal cell loss as compared to WT, again suggesting a protective role of APP in TBI (Corrigan et al., 2012a). Once again, posttraumatic application of exogenous APPsα mitigated these deficits (Corrigan et al., 2012b). These protective effects were found to be mediated by the heparin-binding D1 and D6a domains of APPsα (Corrigan et al., 2011). In an additional study conducted by the same group, the neuroprotective site was pinned down to the APP96-110 sequence in D1, which, applied intraventricularly post-trauma, was enough to significantly improve histological and functional outcome (Corrigan et al., 2014).

At first glance, these findings seem to contradict exacerbation of amyloid pathology and increased risk of AD following TBI. However, there are several possibilities how the two mechanisms may be reconciled. First, APPsα may exert neuroprotective functions independent from the detrimental effects of Aß or amyloid plaques. Second, APPsα could prevent deposition of Aß and further growth of plaques. Third, APPsα may promote clearance of plaques. There is evidence for all three mechanisms. APPsα was shown to modulate BACE activity, possibly inhibiting amyloidogenic cleavage (Obregon et al., 2012). As described in sections “APPsα and APPsβ” and “Mechanisms of Neuroprotection by APP and APPsα Protection in Hypoxia-Ischemia, Excitotoxicity, Degeneration”, APPsα counteracts Aß-mediated excitotoxic damage and delayed degeneration by various trophic and regulatory effects on calcium homeostasis, synaptic function and survival pathways. Recently Fol et al. (2016) discovered that APPsα can ameliorate amyloid pathology by recruitment of microglia, underlining its involvement in clearance of amyloid.

## Brain Ischemia, APP and AD

### Over-Expression, Amyloidogenic Processing of APP and Increased Risk of AD

Cardiovascular diseases and ischemic stroke share overlapping genetic and metabolic risk factors such as hypertension, dyslipidemia, glucose intolerance or diabetes and adipositas (Arboix, 2015). Recently these risk factors were established to also increase the odds of developing AD (Orehek, 2012; Wiesmann et al., 2013; Traylor et al., 2016). Moreover, hypoxic-ischemic conditions of the brain such as in ischemic stroke (Honig et al., 2003), heart arrest (de la Torre, 2006), and cerebral small vessel disease (Cai et al., 2015) directly correlate with AD risk, suggesting that cerebrovascular dysfunction is one possible cause of the neurodegenerative disease (Humpel, 2011; Orehek, 2012). Data from a large meta-analysis (Zhou et al., 2015) and a longitudinal study with over 6500 participants (Tosto et al., 2016) show that ischemic stroke increases AD risk by about 1.6 to 2.2-fold, respectively. Several studies indicate that, vice versa, AD patients have an increased risk to develop ischemic (Chi et al., 2013) and hemorrhagic (Chi et al., 2013; Tolppanen et al., 2013; Zhou et al., 2015) stroke and have a higher prevalence of cerebrovascular lesions (Jellinger, 2010). Other studies, however, did not find an increased risk of ischemic stroke in patients with AD (Imfeld et al., 2013; Tolppanen et al., 2013; Zhou et al., 2015). Not surprisingly, cerebrovascular disease and AD contribute additively to cognitive impairment in patients (Hohman et al., 2015) and mouse models (Pimentel-Coelho et al., 2013), possibly forming a vicious cycle of ischemia and neurodegeneration (Pluta et al., 2013).

It has been suggested that cerebrovascular disease, vascular dementia and AD share common pathophysiological cascades such as altered APP processing (Selnes et al., 2010), perturbed energy metabolism (Chen and Zhong, 2013) and pathological immune response (Brod, 2000). These common pathways may then result in overlapping histopathological findings (de la Torre, 2002; Pluta et al., 2009, 2012; Attems and Jellinger, 2014). APP overexpression and Aβ deposition likely play a pivotal role in these processes. In ischemic stroke patients, expression of APP is indeed increased (Pottier et al., 2012) and serum Aβ levels are elevated, correlating with infarct size and clinical outcome (Lee et al., 2005). Likewise, patients who suffered from hypoxia during a cardiac arrest present with increased Aβ levels, which—again—correlate with clinical outcome (Zetterberg et al., 2011). Increased age-related deposition of Aβ was also shown in chronic cerebral vascular disease in rats (Schreiber et al., 2014). However, elevation of Aβ following ischemia is transient. A recent study employing Pittsburgh Compound-B positron emission tomography (^11^C-PiB-PET; an *in vivo* imaging method of amyloid), revealed no accumulation of Aβ in patients 18 months after ischemic stroke (Sahathevan et al., 2016).

### Neuroprotective Role of APP in Ischemia in Animal Studies

At the first glance, findings concerning APP in conditions of hypoxia/ischemia seem to be contradictory. On the one hand, the pathological role of APP is supported by multiple animal studies. On the other hand, several studies show beneficial effects of APP in animal models of hypoxia-ischemia. It can be assumed that these opposing effects are mediated by the different cleavage products of APP.

On the one hand, ischemia and oxidative stress enhance BACE-1 and γ-secretase activity, resulting in increased Aβ deposition in rats and mice (Sun et al., 2006; Guglielmotto, 2009; Li et al., 2009). APP accumulates in regions of neurodegeneration following focal cerebral ischemia in the rat (Stephenson et al., 1992). Stroke in rats with Aβ pathology leads to aggravated comorbidity, hippocampal atrophy, and cognitive impairment, similar to the consequences of stroke in AD patients (Amtul et al., 2014).

On the other hand, postischemic intraventricular application of APPsα increases neuronal survival in a model of transient focal ischemia in rats (Smith-Swintosky et al., 1994). APP-KO as well as BACE-KO mice are unable to maintain cerebral blood flow and experience drastically increased acute mortality in a model of global cerebral ischemia (Koike et al., 2012). Overexpression of APP provides neuroprotection following middle cerebral artery occlusion in rats (Clarke et al., 2007). There is compelling evidence that APP acts as a potent anti-thrombotic agent (Van Nostrand, 2016). Moreover, it is required for effective immune and glial cell responses to inflammatory stimuli (Carrano and Das, 2015). With glutamate excitotoxicity being a key pathomechanism of ischemic neuronal damage (Broughton et al., 2009), activation of ADAM10, and thus facilitation of APPsα production, provides neuroprotection against excitotoxic stress *in vivo* (Clement et al., 2008). These findings are in line with models of AD where expression of APPsα protects against neurodegeneration and rescues synaptic function (Fol et al., 2016).

Taken together, these data support the importance of balance between the beneficial APPsα and the neurotoxic amyloidogenic pathway, thus resolving the initially contradictive results.

## Mechanisms of Neuroprotection by APP and APPsα Protection in Hypoxia-Ischemia, Excitotoxicity, Degeneration

As outlined in previous sections, ischemia, traumatic injury and degeneration share some common pathological cascades leading to neuronal death (see also Figure 1). One common mechanism of damage is dysregulation of calcium homeostasis (Mattson et al., 1993b; Webster et al., 2006). Intracellular calcium levels at rest are around 100 nM, and fluctuations in cytosolic calcium concentration are tightly coupled to metabolic and synaptic activity (Berridge et al., 2003). Neuronal calcium homeostasis is disturbed in AD, with strong evidence pointing towards a pivotal role of Aβ in destabilizing the balance between mechanisms increasing and decreasing free intracellular calcium (Khachaturian, 1994; LaFerla, 2002; Green and LaFerla, 2008; Berridge, 2010). Similarly, TBI as well as ischemic-hypoxic insults lead to drastic elevations of cellular calcium of up to 20 μM (Yao and Haddad, 2004; Sun et al., 2008). Such acute, strong increases, as well as longer-lasting mild perturbations of calcium levels initiate a plethora of pathological cascades and can, finally, activate caspases and initiate apoptosis (Mattson and Chan, 2003; Orrenius et al., 2003).

**Figure 2:**
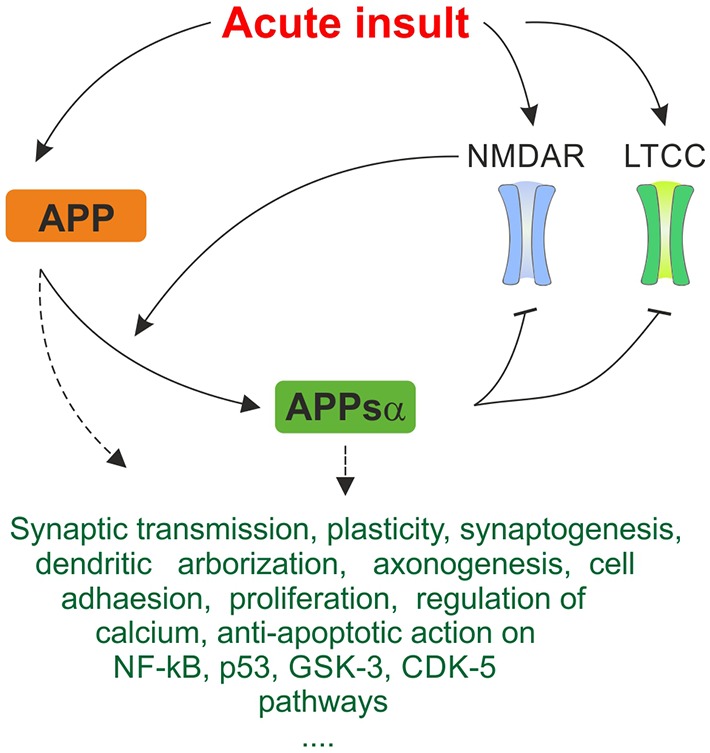
**Simplified summary of proposed neuroprotective mechanisms of amyloid precursor protein (APP) and APPsα in response to acute stress.** Expression of APP is upregulated in response to acute metabolic insult. As depicted in Figure 1, NMDAR and LTCC are pathologically activated, promoting excitotoxic cellular damage. Cleavage of APP is activity-dependent and α-secretases are stimulated by NMDAR, generating the neuroprotective APPsα fragment. APPsα acts inhibitory on NMDAR and LTCC. This negative feedback mechanism may breach the vicious cycle of excitotoxicity and constitute an important protective mechanism in response to acute insults. Several further trophic, regulatory and anti-apoptotic functions of APP and APPsα are listed. They may contribute to acute neuroprotective effect on multiple levels. Since exact mechanisms of interaction are oftentimes not known, this ambiguity is represented by dashed arrows. The triple period below indicates that the list makes no claims of being complete since many more mechanisms are being discussed.

APP and its metabolites, most of all APPsα, intervene with these cascades on multiple levels and exert neuroprotective effects under various conditions of cellular stress, revealing novel possible therapeutical leverage points (Kögel et al., 2012). APPsα was shown to mediate neuroprotection and stabilize intracellular calcium levels in *in vitro* models of excitotoxicity (Mattson et al., 1993a; Ma et al., 2009). The secreted form of APP also protects against Aβ-mediated toxicity in rat hippocampal cell cultures by attenuating Aβ-mediated calcium elevation (Goodman and Mattson, 1994). In a recent study on acute hippocampal slices (Hefter et al., 2016) we showed that APP protects neuronal function in acute hypoxia and promotes recovery of neuronal activity. The protective effects were largely exerted by the APPsα fragment and mediated by inhibition of L-type calcium channels (LTCC). These voltage-gated calcium channels (VGCC) are beside other calcium-permeable membrane channels such as NMDA receptors (NMDAR) and internal stores major sources of intracellular calcium (Yao and Haddad, 2004; Thibault et al., 2007), thus contributing to traumatic/ischemic neuronal damage as well as to the pathophysiology underlying AD. Figure 2 summarizes major neuroprotective mechanisms of APP and APPsα as discussed below.

### Modulation of NMDA Receptors

Traumatic and ischemic injury is marked by aberrant neuronal activity and excessive glutamate release from neurons and glia, mediating excitotoxicity through enhanced activation of glutamate receptors including the calcium-permeable NMDAR. These processes form a vicious cycle of excessive cation influx, further depolarization, opening of more channels and, eventually, breakdown of the membrane potential, osmotic cell swelling and death (Broughton et al., 2009; McAllister, 2011; see also Figure 1). Application of NMDAR blockers is an established neuroprotective strategy in models of excitotoxicity, hypoxia-ischemia and TBI (Kubo et al., 2001) models. Remarkably, APPsα suppresses NMDAR-mediated currents (Furukawa and Mattson, 1998), potently attenuating calcium responses and thus providing protection against NMDAR-mediated excitotoxicity in hippocampal cell culture (Furukawa et al., 1996; Furukawa and Mattson, 1998; Figure 2). Seemingly contradicting these results, APPsα was shown to enhance LTP in acute hippocampal slices (Ring et al., 2007) as well as *in vivo*, where intrahippocampal application of the protein increased NMDAR currents, rescued LTP and memory performance (Taylor et al., 2008). This apparent discrepancy may be due to activation of different NMDAR subtypes which, dependent on their subcellular localization (synaptic vs. extrasynaptic) may promote either synaptic potentiation or proapoptotic effects (Hardingham et al., 2002; von Engelhardt et al., 2007). In our experiments slices from wildtype mice showed postischemic potentiation of evoked population responses whereas synaptic transmission in slices from APP-KO mice was drastically reduced (Hefter et al., 2016). As this kind of plasticity also depends on NMDAR (Maggio et al., 2015), it may also be modulated by APP.

However, we could not observe involvement of NMDAR in APP-mediated protection from hypoxia. Taking into account that secretion of APPsα is activity-dependent (Kirazov et al., 1997; Gakhar-Koppole et al., 2008), its effects on NMDAR may provide a negative-feedback loop on extrasynaptic NMDAR in excitotoxicity or a positive feedback loop on subsynaptic NMDAR in LTP and learning.

### Modulation of L-Type Calcium Channels

LTCC belong to the family of VGCC, which are—depending on the membrane potential—almost exclusively conductive for calcium (Zuccotti et al., 2011). They are one of the major sources of extracellular calcium influx in ischemia (Cataldi, 2013) and contribute to neurodegeneration in AD when over-activated by Aβ (Webster et al., 2006). The subtype Ca_v_1.2 has been identified as a potential pharmacotherapeutical target (Anekonda and Quinn, 2011). Several studies point towards beneficial effect of LTCC blockers in AD patients (Anekonda and Quinn, 2011; Lovell et al., 2015) as well as in animal models of ischemia and neurodegeneration (Gholamipour-Badie et al., 2013). APP was shown to interact directly with Ca_v_1.2 in cultured hippocampal and striatal inhibitory interneurons, with lack of APP resulting in aberrant activity of Ca_v_1.2 and altered short-term plasticity (Yang et al., 2009). In primary cultures of rat cortical neurons expression of human APP inhibited calcium oscillations by modulation of LTCC, suggesting a pivotal role in control of neuronal excitability (Santos et al., 2009). In line with these results we recently found an important role of LTCC for APP-mediated neuroprotection in hypoxia (Hefter et al., 2016). These studies suggest that regulation of LTCC function and thereby cytosolic calcium levels by APP may be neuroprotective. However, the molecular mechanisms underlying regulation of LTCC by APP, the function in healthy neurons and the role in ischemia and degeneration remain elusive.

### Effect of APP on Intracellular Calcium Stores

Internal calcium stores, most importantly the ER and mitochondria, play a major role in the regulation of intracellular calcium homeostasis and contribute to elevations of calcium levels under pathological conditions (Mattson et al., 2000). Regulation of store-related calcium homeostasis appears to be mediated by the intracellular domain of APP, i.e., AICD. In cell culture studies, AICD-deficient cells show increased cytosolic calcium concentrations, decreased ability of the ER to buffer calcium and decreased levels of ATP (Hamid et al., 2007). Although a direct binding of AICD to ER receptors such as ryanodine or inositol triphosphate (IP3) receptors has not been described, indirect effects on ER stores and on calcium signaling in general are discussed. One such mechanism is modulation of phosphoinositide-regulated signaling by regulation of the PIKfyve complex, an essential kinase that synthesizes phosphatidylinositol-3,5-bisphosphate. Its loss of function results in neurodegeneration (Balklava et al., 2015; Currinn and Wassmer, 2016). APP was also proposed to modulate IP3 by affecting the transcription of GSK 3b (Hamid et al., 2007). Moreover, AICD may affect calcium levels by binding to X11, BP1, ShcA and other adaptor proteins which might link it to calcium signaling pathways (LaFerla, 2002) and regulate the expression of genes involved in calcium homeostasis such as S100a9 (Leissring et al., 2002; Pardossi-Piquard and Checler, 2012).

### Effect on Survival/Apoptosis Signaling Pathways and Gene Expression

In severely compromised tissue, such as the ischemic core in stroke, neurons undergo necrosis due to osmotic swelling, lack of energy metabolites and breakdown of ion gradients (Lo et al., 2003). Under milder and longer-lasting metabolic stress, such as in the ischemic penumbra zone or in chronic cerebral hypoperfusion, the balance between anti- and proapoptotic pathways inclunding NF-κB and p53-pathways may tilt towards apoptotic death (Dirnagl et al., 1999; Broughton et al., 2009). In studies on cultured cells, APPsα was shown to exert anti-apoptotic effects by mechanisms such as upregulation of immediate early gene transcription factors, activation of CREB and NF-κB, genes related to cell survival (Guo et al., 1998; Ryan et al., 2013), phosphorylation of glycogen synthase kinase 3β (GSK-3β; Jimenez et al., 2011) or regulation of expression of cyclin-dependent kinase 5 (CDK-5; Hartl et al., 2013). Effects may be mediated by binding of APPsα to several different receptor proteins which are not yet unambiguously identified (Gustafsen et al., 2013). Potential targets include membrane-bound APP itself (Milosch et al., 2014), and direct inhibition of BACE-1 by APPsα which would counteract Aβ-mediated neurotoxicity (Obregon et al., 2012). Further protection of amyloid toxicity by APPsα was mediated by increased expression of the neuroprotective proteins transthyretin and insulin-like growth factor 2 and subsequent inhibition of the proapoptotic BAD (Stein et al., 2004). AICD was described to interact with more than 20 adaptor proteins including Fe65 proteins members of the Mint/X11 family and members of the JIP family (c-jun-N-terminal kinase interacting protein, JIP1b and JIP2), translocate to the nucleus and interact with survival-related genes (Pardossi-Piquard and Checler, 2012).

### Effects on Neurogenesis and Proliferation

Recent years have provided evidence for neurogenesis in several regions of the adult human central nervous system including the dentate gyrus, striatum and olfactory bulb. This mechanism can, in principle, enhance cognitive functions and support recovery from neuronal damage (Inta and Gass, 2015). APPsα was found to stimulate proliferation of neuronal progenitor cells in the subventricular zone (Caillé et al., 2004) and in the hippocampus (Baratchi et al., 2012), whereas AICD was reported to have antiproliferative effects (Zhou et al., 2011). An imbalance between these APP products and thus between neurogenesis and degeneration may contribute to the development of AD (Zhou et al., 2011). Recently APP was shown to control adult hippocampal neurogenesis through GABAergic interneurons, regulating GABAergic synaptic transmission (Wang et al., 2014). APP’s known trophic effects on neuronal viability, cell adhesion, axonogenesis, dendritic arborization and dendritic spines may also contribute to recovery from traumatic and metabolic insults and counteract degeneration (Perez et al., 1997; Lee et al., 2010). APPsα-mediated trophic effects are activity-dependent and stimulated by activation of 5-HT_4_ and NMDAR (Gakhar-Koppole et al., 2008; Cochet et al., 2013), suggesting them to be a feasible adaptive strategy in LTP, plasticity and excitotoxicity.

## Potential Therapeutic Strategies

**Figure 3:**
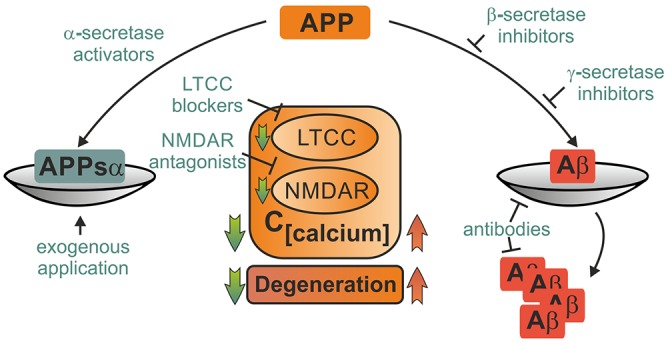
**Potential therapeutical interventions targeting APP metabolites and its binding partners.** Several strategies picking up on involvement of APP and its metabolites in pathophysiological mechanisms following acute insults are briefly portrayed. Depiction as a scale emphasizes the importance of balanced APP metabolism. Therapeutical strategies may employ either enhancement of neuroprotective action of APPsα such as block of LTCC and NMDAR or elevation of APPsα levels by activation of α-secretases or exogenous application. Other approaches might aim at mitigating harmful effects of amyloid ß either by inhibition of its production, prevention of deposition into plaques or facilitated degradation. These strategies might ameliorate acute damage as well as prevent further degeneration.

Currently available pharmacological therapies in AD are mostly based on acetylcholine esterase inhibitors such as donepezil and rivastigmine or NMDAR blockers like memantine. They are far from eliminating the (unknown) primary cause of the disease, but do only alleviate symptoms and delay disease progression (Huang and Mucke, 2012). Likewise, immunotherapeutic approaches with antibodies against Aβ reduce amyloid burden, but show only limited success in the prevention of cognitive decline in ongoing phase III clinical trials (Reiman, 2016). One reason for the slow and tedious progress in therapy of AD may be that cognitive deficits in AD arise not only due to an excess of toxic metabolites, but also from loss of function of protective APP products. Likewise, the past two decades of research on neuroprotective strategies in ischemic stroke and TBI have been hampered by failures to translate results from bench to bedside (Hoyte et al., 2004; O’Collins et al., 2006). Mechanistic understanding of APP’s role in these diseases may help to break this streak. Restitution of the perturbed balance between harmful and beneficial APP metabolites emerges as a promising neuroprotective strategy. Although the hope to find a “cure for all” seems delusive, shared pathological mechanisms in ischemia, injury and AD imply that discovery of common leverage points for novel drugs may be feasible. We will briefly discuss such potential therapeutic strategies which may comprise activation of α-secretases, inhibition of ß- and γ-secretases, exogenous administration of APPsα or amyloid antibodies, control of cellular calcium levels by block of LTCC and NMDAR, activation of neuroprotective mechanisms and inhibition of proapoptotic downstream targets of APP (Figure 3; Selkoe, 2011).

### Activation of α-Secretases

A variety of potential strategies may shift the balance towards non-amyloidogenic cleavage of APP, including modulation of expression, trafficking and regulation of ADAM10 (Postina, 2012). Direct activation of α-secretases by etazolate has been shown to be beneficial in TBI in mice (Siopi et al., 2013). As an indirect mechanism, activation of muscarinic M1 acetylcholine receptors has been reported to increase α-secretase cleavage of APP and decrease Aß levels (Beach et al., 2001). Another activator of α-secretase and inhibitor of β- und γ-secretase is melatonin (Mukda et al., 2016). Increased dimerization of APP via specific compounds such as disulfiram was shown to shift the balance towards non-amyloidogenic cleavage products of APP and thus may present a novel therapeutic approach (Libeu et al., 2012). However, overexpression or activation of ADAM-10 may also have harmful consequences due to effects on other substrates of this enzyme (Clement et al., 2008).

### Inhibition of β- and γ-Secretases

In mice, a selective γ-secretase inhibitor has already been successfully tested, reaching a 33% reduction of Aβ levels within 1 week, without causing severe side effects (Basi et al., 2010). In a mouse model of TBI, pharmacological inhibition of γ-secretase activity reduced post-traumatic tissue loss and improved motor and cognitive recovery (Loane et al., 2009). Both γ- and β-secretase process various other substrates than APP, complicating the use of respective inhibitors (John et al., 2003). However, strategies to specifically inhibit APP cleavage by BACE-1 do exist (Ben Halima et al., 2016) and first BACE-1 inhibitors made it into clinical trials (Vassar et al., 2014).

### Regulation of Calcium Homeostasis

Regulation of intracellular calcium is a promising neuroprotective strategy (Duncan et al., 2010). As discussed above, APP stabilizes calcium homeostasis by interacting with LTCC, NMDAR and other signaling pathways, offering some feasible pharmacological leverage points. LTCC blockers of the dihydropyridine family such as the common antihypertensive drugs nimodipine and nifedipine attenuate progression of dementia in humans, inhibit Aβ formation in cell culture (Lovell et al., 2015), counteract Aβ-mediated calcium increase and excitotoxicity (Anekonda and Quinn, 2011) and alleviate Aβ-related memory deficits in animal models (Gholamipour-Badie et al., 2013). The NMDAR blocker memantine is not only an established drug for treatment of AD (Danysz and Parsons, 2012), but has also protective effects against excitotoxicity in small doses, being potentially beneficial in patients with high risk of ischemic stroke (Trotman et al., 2015). Other potential calcium-stabilizing approaches target downstream pathways of AICD (Nagase and Nakayama, 2014).

### Delivery of Exogenous APPsα

As proven in rodent models of TBI, intraventricular application of exogenous APPsα or its heparin binding domain promote neuronal survival and improve functional outcome (Corrigan et al., 2014). Following TBI or malignant stroke, patients often receive a decompressive craniotomy including ventricular drainage or insertion of an intracranial pressure probe. Application of APPsα through these entries seems feasible. However, these results are highly preliminary and it remains to be proven whether this technique is safe, beneficial and technically feasible in human patients.

## Conclusion

The immense multitude and complexity of APP interactions and functions discovered in recent decades may seem overwhelming and evoke the concern to miss the forest for the trees. Nevertheless, some common principles have emerged from recent studies. APP is more than the mother molecule of amyloid, and AD is more than an amyloido-tauopathy. In this review article, we present convergent evidence from human studies, animal models and *in vitro* experiments for a neuroprotective role of APP in ischemia, brain injury and neurodegeneration. Most studies suggest that these neuroprotective and trophic effects are mainly conducted by the extracellularly secreted fragment APPsα, whereas amyloidogenic cleavage leads to various harmful consequences. We hypothesize that under pathological conditions the cleavage balance of APP is disturbed, and loss of its neuroprotective function may contribute to disease development. While the pathological role of APP in AD may result from an overshoot of pathological products of APP (Aß), production of the neuroprotective soluble fragment APPsα may, in turn, reflect the normal, beneficial reaction of the organism to metabolic challenges. Therefore shifting this balance towards APPsα secretion may be a promising treatment strategy in AD, stroke and TBI. Causal treatments are urgently needed in these conditions. Novel therapeutic targets arise from unraveling the mechanisms of APP-mediated neuroprotection such as regulation of cellular calcium levels by LTCC and NMDAR inhibition, regulation of survival and apoptosis signaling pathways, trophic effects on synapto- and neurogenesis, synaptic function, plasticity and memory formation. However, current understanding of these highly complex processes and the specific contributions of APP is far from complete, and successful translation into clinic is still a major challenge. One of the reasons might be the predominant focus on histopathological endpoints in most studies in the field, largely neglecting longitudinal functional studies. Deeper comprehension of APP-related processes in living tissue, employing functional electrophysiological and imaging techniques should complement morphological studies. Combined (interventional) functional and structural evidence may help to develop new neuroprotective therapies.

## Author Contributions

DH and AD designed, drafted, wrote and revised this work and approved this version to be published. DH designed and created the figures.

## Funding

This study was supported by the DFG Research Group 1332.

## Conflict of Interest Statement

The authors declare that the research was conducted in the absence of any commercial or financial relationships that could be construed as a potential conflict of interest.
